# Author Correction: Mothers with higher twinning propensity had lower fertility in pre-industrial Europe

**DOI:** 10.1038/s41467-022-34954-7

**Published:** 2022-11-25

**Authors:** Ian J. Rickard, Colin Vullioud, François Rousset, Erik Postma, Samuli Helle, Virpi Lummaa, Ritva Kylli, Jenni E. Pettay, Eivin Røskaft, Gine R. Skjærvø, Charlotte Störmer, Eckart Voland, Dominique Waldvogel, Alexandre Courtiol

**Affiliations:** 1 Department of Anthropology, Durham University, Durham, UK; 2 Department of Evolutionary Genetics, Leibniz Institute for Zoo and Wildlife Research, Berlin, Germany; 3 Institut des Sciences de l’Évolution (ISEM), Université de Montpellier, CNRS, EPHE, IRD, Montpellier, France; 4 Center for Ecology and Conservation, University of Exeter, Penryn, UK; 5 Department of Social Research, University of Turku, Turku, Finland; 6 Department of Biology, University of Turku, Turku, Finland; 7 Department of History, University of Oulu, Oulu, Finland; 8 Department of Biology, Norwegian University of Science and Technology, Trondheim, Norway; 9 Institute for Philosophy, Justus Liebig University Gießen, Giessen, Germany; 10 Department of Evolutionary Biology and Environmental Studies, University of Zurich, Zurich, Switzerland

**Keywords:** Biological anthropology, Behavioural ecology, Human behaviour, Statistical methods

Correction to: *Nature Communications* 10.1038/s41467-022-30366-9, published online 24 May 2022

In this article the funding from Deutsche Forschungsgemeinschaft (DFG, German Research Foundation) – project number 491292795 was omitted. The original article has been corrected.

