# Supplementary material for: Mothers with higher twinning propensity had lower fertility in pre-industrial Europe
Source: Nat Commun. 2022 May 24;13:2886. doi: 10.1038/s41467-022-30366-9 (PMC9130277; doi:10.1038/s41467-022-30366-9)
Supplement: Supplementary file 1 — Supplementary Information [file 41467_2022_30366_MOESM1_ESM.pdf]

Supplementary Information for

Mothers with higher twinning propensity had lower fertility in pre-industrial Europe

I. J. Rickard   C. Vullioud   F. Rousset   E. Postma   S. Helle
V. Lummaa   R. Kylli   J. E. Pettay   E. Røskaft   G. R. Skjærvø
C. Störmer   E. Voland   D. Waldvogel   A. Courtiol*

Contents

1 Supplementary Notes **2**

2 Supplementary Figures **4**

3 Supplementary Tables **10**

*corresponding author: courtiol@izw-berlin.de

1 Supplementary Notes

We face the problem of testing Goodness-Of-Fit (GOF) when the distribution of the test statistic depends on fitted parameters, and we use a bootstrap procedure for that purpose. While this practice is widespread, it is widely ignored beyond the theoretical literature that this may be incorrect, so we first recall some theory and then consider corrections for this procedure.

We aim to assess whether the slope $s = \mathrm{s}(\mathcal{D})$ we estimate from the actual data $\mathcal{D}$ by the logistic regression (model 3; see Methods) between the per-birth twinning probability and maternal total births is consistent with the distribution $\phi(S = \mathrm{s}(D); \boldsymbol{\theta})$ of slopes expected for samples D drawn under a given hypothetical scenario π (say "P") with parameters $\boldsymbol{\theta}$. We do not know the $\boldsymbol{\theta}$ vector so we estimate it from the data, and the estimates depend on the assumed π (i.e., we obtain different estimates $\hat{\boldsymbol{\theta}}_\pi$ for π="P", "I", and so on). Thus we use the slope as a GOF statistic for a model with parameters $\boldsymbol{\theta}$ estimated aside of the slope, on the same data.

For a GOF test, we can then simulate the distribution $\phi(S; \hat{\boldsymbol{\theta}}_\pi)$ and use this distribution as an approximation for $\phi(S; \boldsymbol{\theta})$. That is, we can obtain by simulation an estimate $\hat{\mathrm{CDF}}(x; \hat{\boldsymbol{\theta}}_\pi)$ of the cumulative distribution function (CDF) of $S = \mathrm{s}(D^*)$ for bootstrap samples D^* generated under $\hat{\boldsymbol{\theta}}_\pi$, i.e., an estimate of $\Pr(S \leq x; \hat{\boldsymbol{\theta}}_\pi)$ for any x, and determine a unidirectional p-value as the value of the estimated CDF value for observed slope, $p = \hat{\mathrm{CDF}}(\mathrm{s}(\mathcal{D}); \hat{\boldsymbol{\theta}}_\pi)$ (or $1 - p$, depending on context). This is what a naive use of the bootstrap would provide.

However, we fitted parameters on the data, which means that the data tend to be more consistent with the fitted model $\phi(S; \hat{\boldsymbol{\theta}}_\pi)$ than with the data-generating process $\phi(S; \boldsymbol{\theta})$. This means that, if a GOF test has controlled error rates (uniform p-values) when defined from $\mathrm{CDF}(x; \boldsymbol{\theta})$, it may be conservative when each sample is assessed against $\mathrm{CDF}(x; \hat{\boldsymbol{\theta}}_\pi)$, with parameters and CDF re-estimated on each sample produced by the data-generating process.

Whether the test is conservative depends on the relationship between the GOF statistic (more generally denoted $T = \mathrm{t}(D)$) and the estimated parameters. A toy example that captures this problem considers that $\boldsymbol{\theta}$ is reduced to a single scalar parameter θ, and that $\hat{\theta}(D)$ follows jointly with T the bivariate Gaussian model

$$\begin{pmatrix} T \\ \hat{\theta} \end{pmatrix} \sim \mathcal{N}\left(\boldsymbol{\mu} = \begin{pmatrix} \beta\theta \\ \theta \end{pmatrix}, \boldsymbol{\Sigma} = \begin{pmatrix} \sigma_t^2 + \beta^2\sigma_{\hat{\theta}}^2 & \beta\sigma_{\hat{\theta}}^2 \\ \beta\sigma_{\hat{\theta}}^2 & \sigma_{\hat{\theta}}^2 \end{pmatrix}\right)$$

with β, σ_t^2 and $\sigma_{\hat{\theta}}^2$ independent of θ. The reason for considering this example is that it leads to a simple linear regression of T to $\hat{\theta}$. Specifically, $T|\hat{\theta}; \theta \sim \mathcal{N}(\beta\hat{\theta}, \sigma_t^2)$,[‡] from which some properties of the naive bootstrap test can easily be deduced. For this test, T is compared to the distribution of T^* values for bootstrap samples from the fitted model (i.e. with $\hat{\theta}$ as parameter). Therefore, for the test to be correct (with a uniform distribution of p-values), the conditional distribution of T given $\hat{\theta}$ should be identical to the simulated distribution of the T^*s. But their variances are different: the conditional variance of T is σ_t^2 (regression result), while the simulation variance is higher ($\sigma_t^2 + \beta^2\sigma_{\hat{\theta}}^2$, this being the same as the variance of T drawn from the distribution with parameter θ). Various forms of non-uniform distributions of p-values may then result; the simplest one occurring when when $\beta = 1$ and σ_t^2 reduces to zero. In that case the means of the two distributions are equal to $\hat{\theta}$, so the distribution of p-values concentrates on 0.5. Since this is true irrespective of the $\hat{\theta}$ value, the p-value will be generally too close to 0.5.

Conservativeness is expected whenever the GOF statistic is positively correlated with one of the parameter estimates. Ideally, one should use a GOF statistic whose distribution is (as least asymptotically) independent of the value of $\hat{\boldsymbol{\theta}}_\pi$, or, equivalently in practice, of the value of $\boldsymbol{\theta}$. In other words, the bootstrap procedure is guaranteed to provide an asymptotically correct GOF test of a statistic T only if T is asymptotically a pivotal statistic under the null model; a statistic being pivotal if its distribution is independent of the value of $\boldsymbol{\theta}$. Using pivotal statistics is also a standard requirement for analytic GOF tests (e.g., [1], §3.3).

Pivotal statistics are not generally available for use in one-step bootstrap procedures. In that case, double bootstrap procedures have been developed (e.g., [2]; [3], p. 177). The basic idea of such procedures is that, for each sample D_b^* from the first bootstrap step, a second bootstrap is performed using bootstrap samples D_{bc}^{**} generated given the estimates $\hat{\boldsymbol{\theta}}_\pi(D_b^*)$ (with $b = 1, \ldots, B$) from the model fit to D_b^*, and $\mathrm{t}(D_b^*)$ can be compared to the distribution of $\mathrm{t}(D_{bc}^{**})$ (with $c = 1, \ldots, C$) providing a value

‡From standard regression theory for gaussian variables: $T|\theta$ has mean $\mathrm{E}[T] + \boldsymbol{\Sigma}_{12}\boldsymbol{\Sigma}_{22}^{-1}(\hat{\theta} - \mathrm{E}(\hat{\theta}))$ and variance $\boldsymbol{\Sigma}_{11} - \boldsymbol{\Sigma}_{12}\boldsymbol{\Sigma}_{22}^{-1}\boldsymbol{\Sigma}_{12}$

$p_b^* = \hat{\text{CDF}}(\text{t}(D_b^*); \hat{\boldsymbol{\theta}}_\pi(D_b^*))$ for each D_b^*. The distribution of p_b^* would be uniform if T were pivotal; instead of assuming that, one considers that the non-uniform distribution of $p = \hat{\text{CDF}}(\text{t}(D); \hat{\boldsymbol{\theta}}_\pi(D))$ is asymptotically pivotal, and obtains an estimate of this distribution as that of p_b^* over different D_b^*, by the double bootstrap calculation.

Double-bootstrap computations are computer intensive, and indeed not practically applicable as described above to the present problem. For such reasons, various simplifications of the above procedure have been discussed in the literature ([3], ch. 9). We apply here the notion of control variates, previously applied for bias correction in non-parametric bootstrap (ibid, Section 9.3; [4]), as follows. A control variate is a variable correlated with T, which can be used to predict its value. The control variate that we consider here is (an estimator of) $\text{E}[S^*; \hat{\boldsymbol{\theta}}_\pi(D)]$, the expected value of the slope from samples drawn under the model fitted to D. The test statistic of the corrected bootstrap test is then the residual of the prediction of the slope $\text{s}(D)$ by the mean value of S^* under $\hat{\boldsymbol{\theta}}_\pi(D)$; the latter mean being obtained by performing a single-level bootstrap simulation. In the literature, simpler control variates have been considered, not requiring a bootstrap simulation; but these previous works considered non-parametric bootstrap simulations, which involve additional assumptions not valid here. In some of these previous works, parameter estimates (elements of $\hat{\boldsymbol{\theta}}_\pi$) clearly correlated with T have been used, in contrast to the present case.

More specifically, we apply the following procedure, which would be exact (apart from finite simulation error) under the toy model. Under the tested model, we draw B replicates of D^* given $\hat{\boldsymbol{\theta}}_\pi(\mathcal{D})$ and C replicates of D^{**} given $\hat{\boldsymbol{\theta}}_\pi(D^*)$. We then perform a *calibration* fit to construct a predictor of $\text{s}(D_b^*)$ from the average value of $\text{s}(D_{bc}^{**})$, by simple regression with intercept and slope. We use the residuals of prediction as GOF statistics. We use $B = 200$ and $C = 49$. We also performed *validation* simulations that demonstrated the need for correcting the single-level bootstrap procedure and the effectiveness of our correction. For this validation, under each of the sixteen scenarios, we simulated $B_\text{v} = 200$ samples under the fitted model and applied to them both the single-level bootstrap test and the testing procedure with control variate. The resulting distributions are shown in Supplementary Figure 7, which confirms the need for a correction for models including mechanisms P and S.

Computationally, the main benefits of using a control variate over the raw double-bootstrap appear (a) when performing a single test, as fewer simulations may be needed to reach a given degree of accuracy in p-value determination; (b) when assessing the performance of the GOF test of an hypothesis π_0 under an alternative model π_1 (and in particular for the validation simulations, where $\pi_0 = \pi_1$). We first perform a double bootstrap simulation to obtain a calibration fit under π_0. Then, for each new sample D generated under π_1, we do not need to perform a new double-bootstrap simulation. Instead, we need only to compute $\text{s}(D)$, $\hat{\boldsymbol{\theta}}_{\pi_0}(D)$, and values of $\text{s}(D^*)$ for C replicates of D^* simulated given $\hat{\boldsymbol{\theta}}_{\pi_0}(D)$, to obtain the residual of prediction of $\text{s}(D)$ from mean $\text{s}(D^*)$ and to compare it to the distribution of residuals from the single double bootstrap simulation on the original D.

Despite the huge savings in computing brought by the control variate approach over a basic double bootstrap, our GOF tests of the different simulation scenarios still have large CPU and memory requirements. Such computations were performed both on a Linux computer with 128 cores and 254 GB of RAM from the Department of Evolutionary Genetics at the Leibniz Institute for Zoo and Wildlife Research (Berlin, Germany), as well as on a Linux cluster node with 112 cores and 3To of RAM of the Meso@LR cluster of the University of Montpellier, requiring a computation time of the order of 10,000 core CPU hours (about half of them for the validation).

2 Supplementary Figures

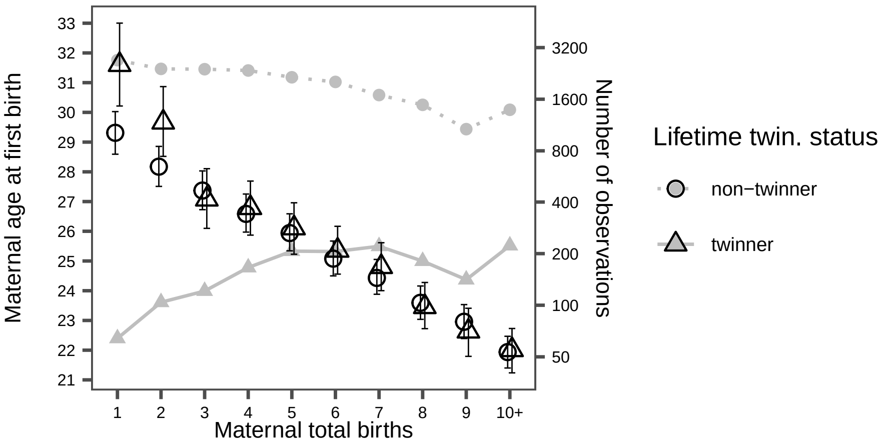

Supplementary Figure 1: Relationship between lifetime twinning status, maternal total births and the age at first birth (n=21,290 mothers in total, from 8 populations; see Table 1 in main text). The age at first birth was significantly higher for twinners with one (AFB_{twinners} = 31.6 years; 95% CI: 30.2, 33.0; $AFB_{\text{non-twinners}}$ = 29.3 years; 95% CI 28.6, 30.0; delay for twinners = 27.5 months; 95% CI: 11.9, 43.7) or two maternal total births (AFB_{twinners} = 29.7 years; 95% CI: 28.6, 30.8; $AFB_{\text{non-twinners}}$ = 28.2 years; 95% CI: 27.5, 28.8; delay for twinners = 17.9 months; 95% CI: 6.55, 29.4). Marginal predictions for the age at first reproduction are shown in black with open symbols. Their values are provided by the left y-axis. The distribution of maternal total births for both twinners and non-twinners is shown by the grey lines with filled symbols, with the numerical value given by the log-scaled y-axis on the right of the figure. 95% CIs are shown as error bars and are based on 1000 parametric bootstrap replicates for each depicted category of maternal total births.

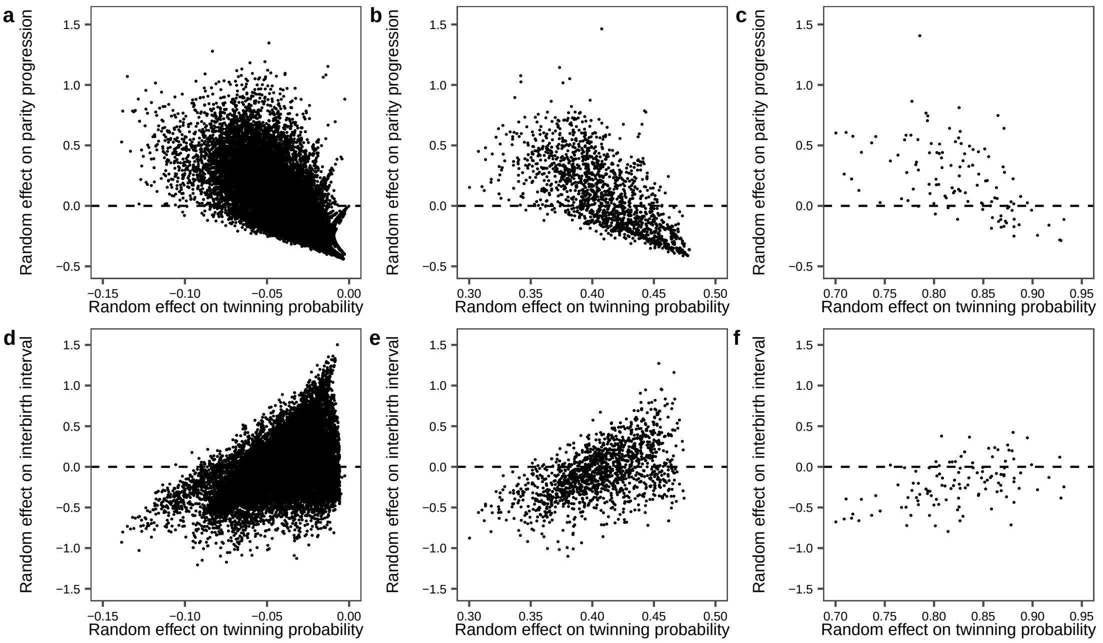

Supplementary Figure 2: Relationships between the random effects for the three life history traits. Top row (A-C) shows the relationship between the random effect from the fit of model 4 (the full model predicting parity progression; see Methods) and 6 (the full model predicting per-birth twinning probability). Bottom row (D-F) shows the relationship between the random effect from the fit of model 5 (the full model predicting the interbirth interval) and 6. Left column (A & D) shows the relationship for mothers that did not produce any twins during their life. Middle column (B & E) shows the relationship for mothers that produced twins once during their life. Right column (C & F) shows the relationship for mothers that produced twins twice during their life. All axes are represented on the scale of the linear predictor of the corresponding models. Relationships for mothers that produced twins more than twice during their life are not displayed but follow similar patterns.

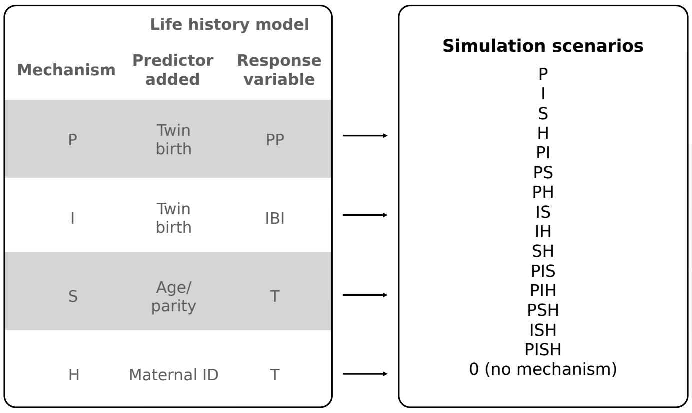

Supplementary Figure 3: Hypothetical mechanisms tested by the simulation of alternative scenarios built around models predicting three life history events: parity progression (PP), interbirth interval (IBI) and twinning status of a given birth (T). The bubble on the left shows how the three life history models that were included in all simulation scenarios were modified to test each of the four hypothetical mechanisms (P, I, S, H; see section Results in main text for description). Each life history model describes the effect of predictors on a particular life history event. By default, each life history model includes an intercept and a random effect referring to the population. All models for PP and IBI also consider the effect of age/parity and maternal identity (see Methods). The bubble on the right shows the comprehensive set of combinations of the four mechanisms that were tested to produce simulation scenarios.

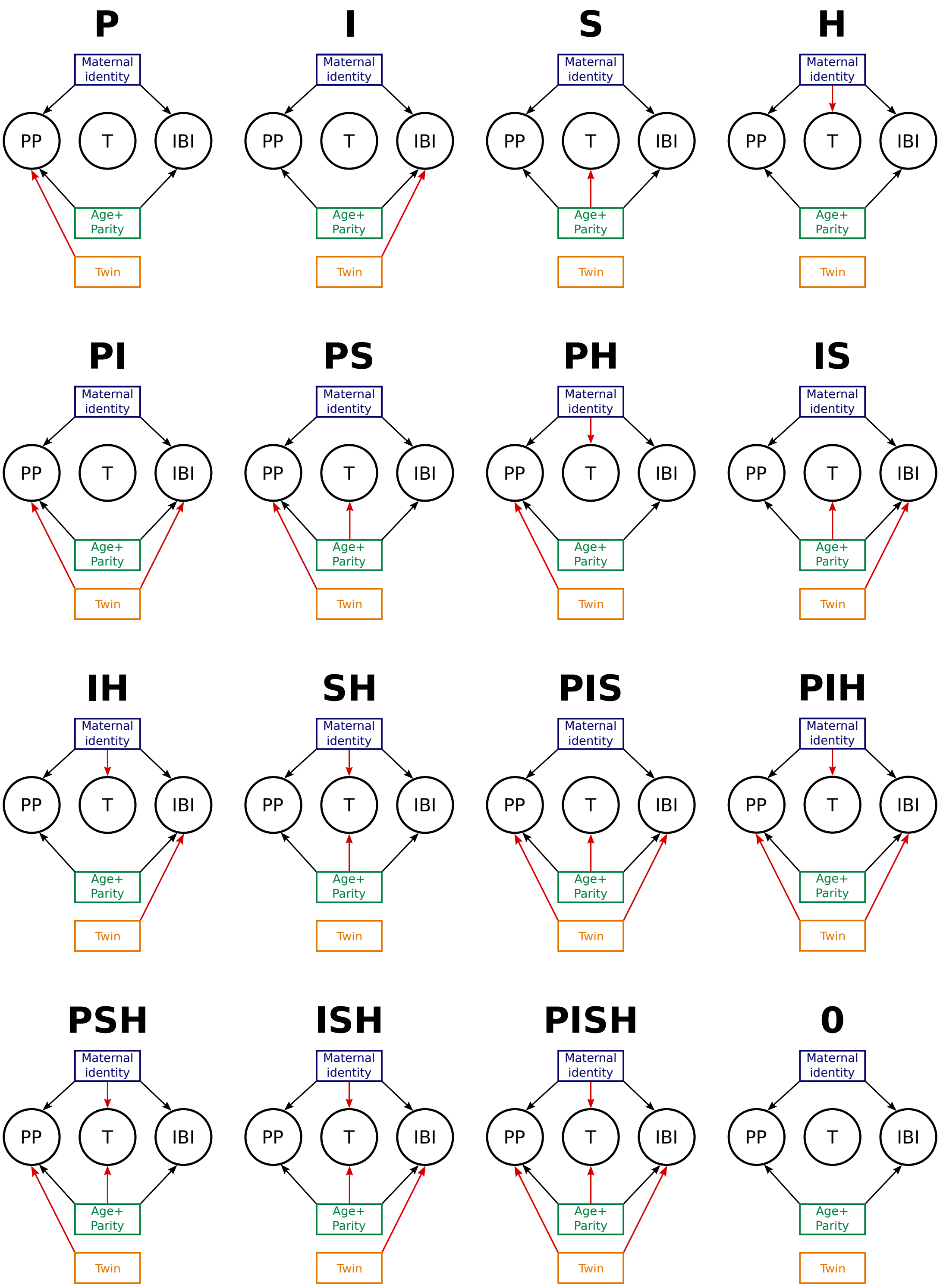

Supplementary Figure 4: Representation of the four mechanisms (row 1) used as the basis for 16 simulation scenarios evaluated (all rows). Circles represent the three life history events we considered: parity progression (PP), probability of twinning (T) and interbirth interval (IBI). The rectangles represent the variables potentially shaping these life history events — maternal age and parity at a given birth (referred to as Age + Parity here; and as `poly(cbind(age, parity), best_order)` in model formulas) and whether the last birth was a twin birth or not (Twin here; `twin` in model formulas) — as well as a random effect capturing other sources of heterogeneity between mothers (Maternal identity here; `maternal_id` in model formulas). Black arrows represent relationships assumed in all simulation scenarios. Another random effect capturing differences between populations was also considered for all life history events and all mechanisms (not shown). Red arrows represent relationships used to activate the mechanisms required by each simulation scenario.

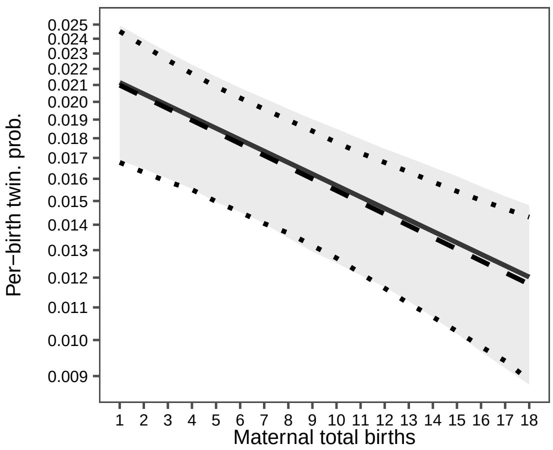

Supplementary Figure 5: Relationship between the per-birth twinning probability and maternal total births (n=23,281 mothers in total, from 9 populations; see Supplementary Table 14). This figure reproduces the relationship shown in main text Fig. 2 (solid line for the regression line and grey area for the 95% CI) but additionally plots the relationship with the inclusion of data from families with missing birth month information (including the entire Norway dataset). This latter relationship is illustrated by the dashed regression line with an estimated slope β' of -0.0346 (95% CI: -0.0511, -0.0183), as well as with the dotted lines showing the location of the 95% CI.

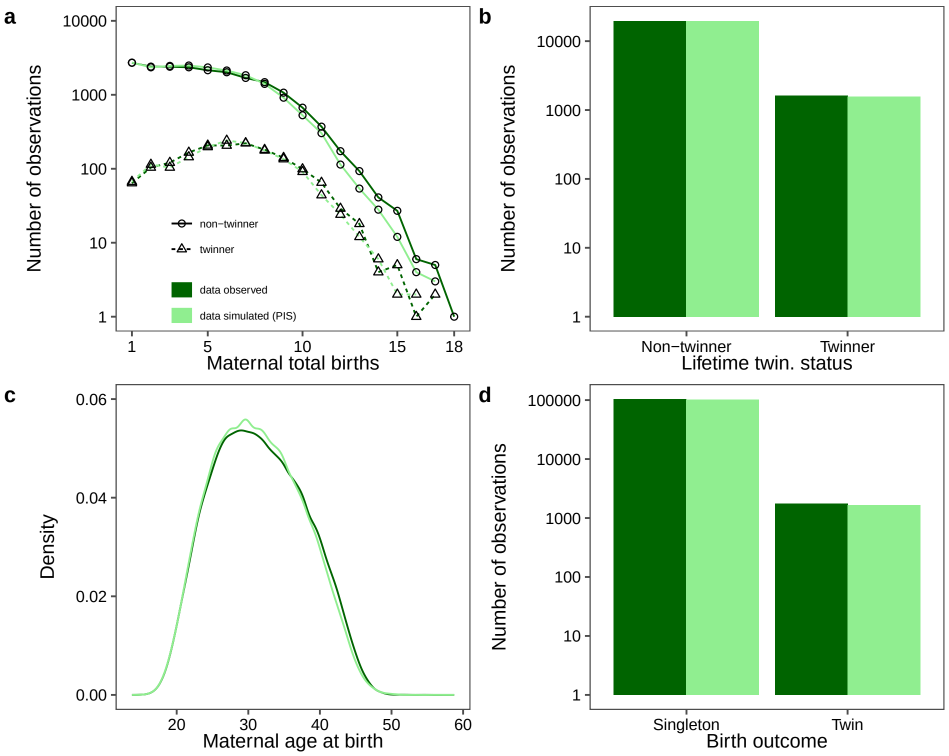

Supplementary Figure 6: Comparison of different metrics between the real data and a dataset simulated under scenario PIS. A: distribution of maternal total births; B: number of twinners and non-twinners; C: distribution of maternal age at birth; D: number of singleton and twin births. The first row (A & B) represents metrics computed at the level of mothers. The second row (C & D) represents metrics computed at the level of births. All these plots show that the simulation scenario PIS produced fertility and twinning data similar to actual observations.

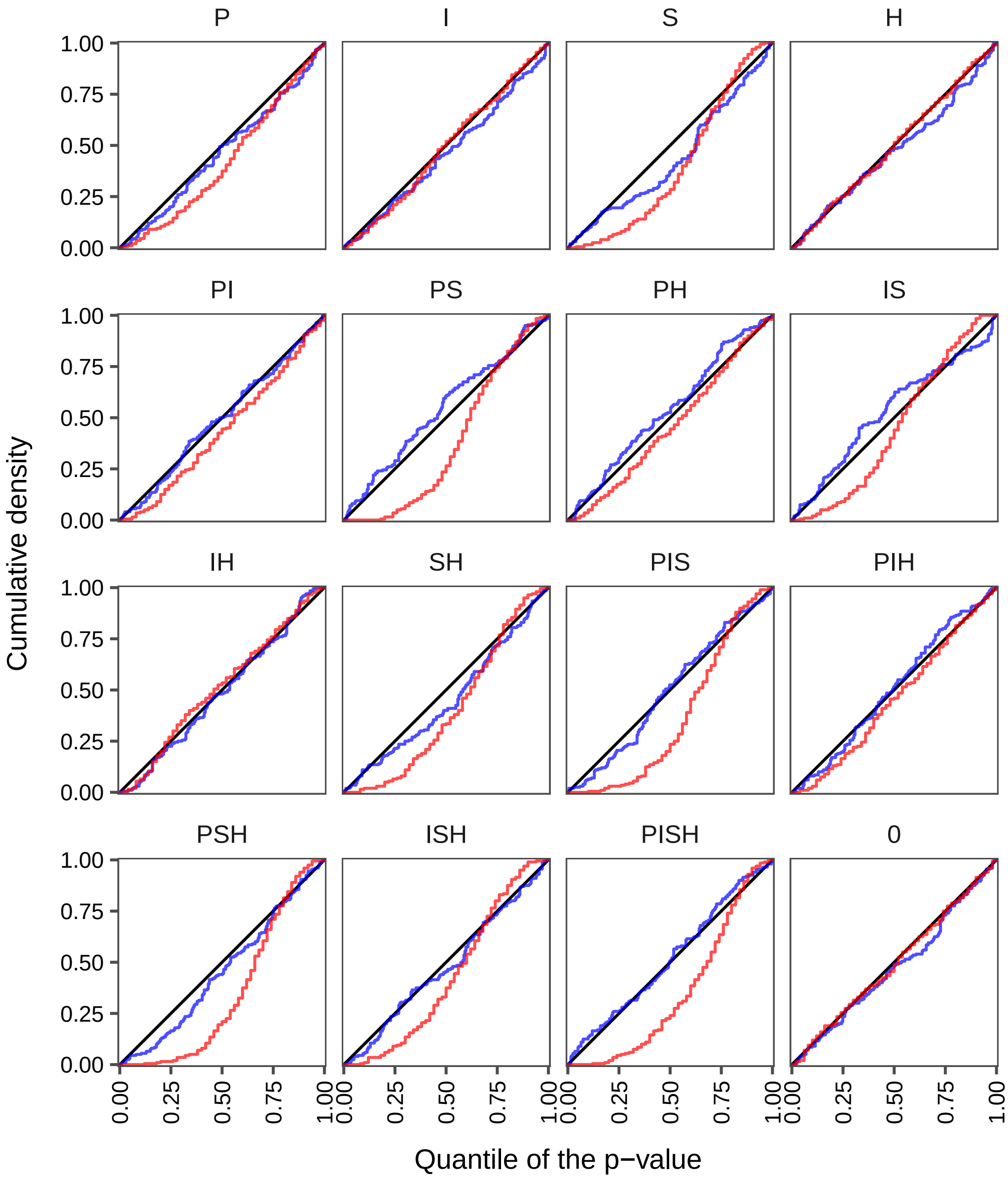

Supplementary Figure 7: Evaluation of the validity of the goodness-of-fit testing procedure. Each plot shows the empirical cumulative distribution of p-values for each tested simulation scenario when the data were simulated under the same scenario. In such a case, the goodness-of-fit test corresponds to the test of the null hypothesis when the null hypothesis is true. Therefore, the probability density of p-values should be uniform and the cumulative distribution should appear close to the straight diagonal line depicted on the plots. However, the empirical cumulative distribution of p-values for the single-level bootstrap (shown in red) clearly departs from this expectation for scenarios including mechanisms P or S, showing that a correction is required. The empirical distributions of the double-bootstrap procedure (shown in blue) corrects this and show that the latter procedure can be applied for all scenarios.

3 Supplementary Tables

Supplementary Table 1: Summary of the fit of model 1. The model fit corresponds to the fit of a model with the following formula: $\texttt{births\_total} \sim 1 + \texttt{twinner} + (1|\texttt{pop})$. In all tables showing the summary of fits, values given in column 3 for fixed effects, random effects (if applicable) and the parameters of the response family (if applicable) are estimates. Other values are the results of computations.

Type	Variable	Value	Cond. SE	t-value
fixed effects	β_1	1.54	0.0319	48.1
	$\beta_{\texttt{twinner}}$	0.279	0.0162	17.3
random effects	variance between pop	0.00785		
response family	truncated negative binomial with log link			
	shape parameter	4.73		
fit info	number of model parameters	4		
	marginal log Likelihood	-50929		
	marginal AIC	101866		
	conditional AIC (cAIC)	101841		
data info	number of fitted observations (N)	21290		

Supplementary Table 2: Summary of the fit of model 2. The model fit corresponds to the fit of a model with the following formula: $\texttt{twinner} \sim 1 + \texttt{births\_total} + (1|\texttt{pop})$. See legend of Supplementary Table 1 for more details.

Type	Variable	Value	Cond. SE	t-value
fixed effects	β_1	-3.35	0.112	-30
	$\beta_{\texttt{births\_total}}$	0.162	0.00855	18.9
random effects	variance between pop	0.0697		
response family	binomial with logit link			
fit info	number of model parameters	3		
	marginal log Likelihood	-5548		
	marginal AIC	11102		
	conditional AIC (cAIC)	11086		
data info	number of fitted observations (N)	21290		

Supplementary Table 3: Summary of the fit of model 3. The model fit corresponds to the fit of a model with the following formula: $\texttt{cbind(twin\_total, singleton\_total)} \sim 1 + \texttt{births\_total} + (1|\texttt{pop})$. See legend of Supplementary Table 1 for more details.

Type	Variable	Value	Cond. SE	t-value
fixed effects	β_1	-3.83	0.104	-36.7
	$\beta_{\texttt{births\_total}}$	-0.0338	0.00864	-3.92
random effects	variance between pop	0.0556		
response family	binomial with logit link			
fit info	number of model parameters	3		
	marginal log Likelihood	-5993		
	marginal AIC	11992		
	conditional AIC (cAIC)	11976		
data info	number of fitted observations (N)	21290		

Supplementary Table 4: Summary of the fit of model 4. The model fit corresponds to the fit of a model with the following formula: $\texttt{PP} \sim 1 + \texttt{twin} + \texttt{poly}(\texttt{cbind}(\texttt{age}, \texttt{parity}), 5) + (1|\texttt{maternal\_id}) + (1|\texttt{pop})$. See legend of Supplementary Table 1 for more details.

Type	Variable	Value	Cond. SE	t-value
fixed effects	β_1	1.57	0.179	8.74
	$\beta_{\texttt{twin}}$	-0.412	0.0636	-6.47
	$\beta_{\texttt{age}}$	-420	59.7	-7.04
	$\beta_{\texttt{age}^2}$	-185	51.7	-3.57
	$\beta_{\texttt{age}^3}$	-43.7	32.6	-1.34
	$\beta_{\texttt{age}^4}$	-15.7	14.3	-1.09
	$\beta_{\texttt{age}^5}$	2.47	9.7	0.255
	$\beta_{\texttt{parity}}$	49.1	97.6	0.502
	$\beta_{\texttt{age}\times\texttt{parity}}$	12922	39528	0.327
	$\beta_{\texttt{age}^2\times\texttt{parity}}$	-33721	30550	-1.1
	$\beta_{\texttt{age}^3\times\texttt{parity}}$	-395	14752	-0.0268
	$\beta_{\texttt{age}^4\times\texttt{parity}}$	-3270	6413	-0.51
	$\beta_{\texttt{parity}^2}$	-18.3	105	-0.175
	$\beta_{\texttt{age}\times\texttt{parity}^2}$	23609	38306	0.616
	$\beta_{\texttt{age}^2\times\texttt{parity}^2}$	-5967	22834	-0.261
	$\beta_{\texttt{age}^3\times\texttt{parity}^2}$	10602	8890	1.19
	$\beta_{\texttt{parity}^3}$	27	64.7	0.417
	$\beta_{\texttt{age}\times\texttt{parity}^3}$	-15417	19086	-0.808
	$\beta_{\texttt{age}^2\times\texttt{parity}^3}$	1259	8611	0.146
	$\beta_{\texttt{parity}^4}$	24.5	21.4	1.14
	$\beta_{\texttt{age}\times\texttt{parity}^4}$	-1816	5027	-0.361
	$\beta_{\texttt{parity}^5}$	-4.69	4.84	-0.97
random effects	variance between `maternal_id`	0.465		
	variance between `pop`	0.104		
response family	binomial with logit link			
fit info	number of model parameters	24		
	marginal log Likelihood	-40429		
	marginal AIC	80905		
	conditional AIC (cAIC)	80276		
data info	number of fitted observations (N)	105833		

Supplementary Table 5: Summary of the fit of model 5. The model fit corresponds to the fit of a model with the following formula: $\texttt{IBI} \sim 1 + \texttt{twin} + \texttt{poly}(\texttt{cbind}(\texttt{age}, \texttt{parity}), 6) + (1|\texttt{maternal\_id}) + (1|\texttt{pop})$. Note that the variable `IBI` fitted in the model actually corresponds to the duration of interbirth interval minus six months. This rescaling prevents numerical issues during the simulations. When this fitted model is used for predictions (in plots or to compute effect sizes), the missing six months are reintroduced to produce correct results. See legend of Supplementary Table 1 for other details.

Type	Variable	Value	Cond. SE	t-value
fixed effects	β_1	3.44	0.0579	59.5
	$\beta_{\texttt{twin}}$	-0.0328	0.015	-2.18
	$\beta_{\texttt{age}}$	-63.6	21.9	-2.9
	$\beta_{\texttt{age}^2}$	39.7	19.6	2.02
	$\beta_{\texttt{age}^3}$	-31.3	13	-2.41
	$\beta_{\texttt{age}^4}$	15	6.31	2.38
	$\beta_{\texttt{age}^5}$	-5	2.42	-2.07
	$\beta_{\texttt{age}^6}$	4.01	1.01	3.95
	$\beta_{\texttt{parity}}$	121	40.7	2.96
	$\beta_{\texttt{age}\times\texttt{parity}}$	-29804	15604	-1.91
	$\beta_{\texttt{age}^2\times\texttt{parity}}$	26630	12833	2.08
	$\beta_{\texttt{age}^3\times\texttt{parity}}$	-15687	7263	-2.16
	$\beta_{\texttt{age}^4\times\texttt{parity}}$	5901	2912	2.03
	$\beta_{\texttt{age}^5\times\texttt{parity}}$	-2056	883	-2.33
	$\beta_{\texttt{parity}^2}$	75.7	54.5	1.39
	$\beta_{\texttt{age}\times\texttt{parity}^2}$	-30418	19139	-1.59
	$\beta_{\texttt{age}^2\times\texttt{parity}^2}$	24308	13414	1.81
	$\beta_{\texttt{age}^3\times\texttt{parity}^2}$	-11197	5960	-1.88
	$\beta_{\texttt{age}^4\times\texttt{parity}^2}$	3100	1618	1.92
	$\beta_{\texttt{parity}^3}$	53	42.1	1.26
	$\beta_{\texttt{age}\times\texttt{parity}^3}$	-17528	12996	-1.35
	$\beta_{\texttt{age}^2\times\texttt{parity}^3}$	9735	7207	1.35
	$\beta_{\texttt{age}^3\times\texttt{parity}^3}$	-2955	2067	-1.43
	$\beta_{\texttt{parity}^4}$	19	19	1
	$\beta_{\texttt{age}\times\texttt{parity}^4}$	-4658	4824	-0.966
	$\beta_{\texttt{age}^2\times\texttt{parity}^4}$	1902	1773	1.07
	$\beta_{\texttt{parity}^5}$	7.84	5.26	1.49
	$\beta_{\texttt{age}\times\texttt{parity}^5}$	-1418	970	-1.46
	$\beta_{\texttt{parity}^6}$	1.68	1.04	1.61
random effects	variance between `maternal_id`	0.163		
	variance between `pop`	0.00375		
response family	negative binomial with log link			
	shape parameter	5.2		
fit info	number of model parameters	32		
	marginal log Likelihood	-337275		
	marginal AIC	674614		
	conditional AIC (cAIC)	663067		
data info	number of fitted observations (N)	84543		

Supplementary Table 6: Summary of the fit of model 6. The model fit corresponds to the fit of a model with the following formula: $\texttt{T} \sim \texttt{1} + \texttt{poly}(\texttt{cbind}(\texttt{age}, \texttt{parity}), \texttt{3}) + (\texttt{1}|\texttt{maternal\_id}) + (\texttt{1}|\texttt{pop})$. See legend of Supplementary Table 1 for more details.

Type	Variable	Value	Cond. SE	t-value
fixed effects	β_1	-4.1	0.113	-36.4
	$\beta_{\texttt{age}}$	73.7	31.1	2.37
	$\beta_{\texttt{age}^2}$	-61.2	17.2	-3.55
	$\beta_{\texttt{age}^3}$	-47.1	16.4	-2.87
	$\beta_{\texttt{parity}}$	-0.936	40.1	-0.0233
	$\beta_{\texttt{age}\times\texttt{parity}}$	-4006	11890	-0.337
	$\beta_{\texttt{age}^2\times\texttt{parity}}$	7710	8669	0.889
	$\beta_{\texttt{parity}^2}$	19	27.3	0.694
	$\beta_{\texttt{age}\times\texttt{parity}^2}$	-4380	8121	-0.539
	$\beta_{\texttt{parity}^3}$	-18.6	14.1	-1.32
random effects	variance between `maternal_id`	0.485		
	variance between `pop`	0.0571		
response family	binomial with logit link			
fit info	number of model parameters	12		
	marginal log Likelihood	-8828		
	marginal AIC	17681		
	conditional AIC (cAIC)	17644		
data info	number of fitted observations (N)	105833		

Supplementary Table 7: Summary of the fit of model 7. The model fit corresponds to the fit of a model with the following formula: $\texttt{AFB} \sim \texttt{1} + \texttt{twinner} * \texttt{births\_total\_fac} + (\texttt{1}|\texttt{pop})$. See legend of Supplementary Table 1 for more details.

Type	Variable	Value	Cond. SE	t-value
fixed effects	β_1	5.86	0.0123	479
	$\beta_{\texttt{twinner}}$	0.0752	0.0207	3.63
	$\beta_{\texttt{births\_total\_fac2}}$	-0.0396	0.00461	-8.6
	$\beta_{\texttt{births\_total\_fac3}}$	-0.0686	0.00462	-14.8
	$\beta_{\texttt{births\_total\_fac4}}$	-0.0977	0.00465	-21
	$\beta_{\texttt{births\_total\_fac5}}$	-0.122	0.00477	-25.6
	$\beta_{\texttt{births\_total\_fac6}}$	-0.156	0.00486	-32.1
	$\beta_{\texttt{births\_total\_fac7}}$	-0.182	0.00513	-35.5
	$\beta_{\texttt{births\_total\_fac8}}$	-0.217	0.00536	-40.5
	$\beta_{\texttt{births\_total\_fac9}}$	-0.244	0.00601	-40.7
	$\beta_{\texttt{births\_total\_fac10+}}$	-0.29	0.00551	-52.6
	$\beta_{\texttt{twinner:births\_total\_fac2}}$	-0.0236	0.0264	-0.894
	$\beta_{\texttt{twinner:births\_total\_fac3}}$	-0.086	0.0258	-3.34
	$\beta_{\texttt{twinner:births\_total\_fac4}}$	-0.0676	0.0246	-2.75
	$\beta_{\texttt{twinner:births\_total\_fac5}}$	-0.0688	0.0239	-2.87
	$\beta_{\texttt{twinner:births\_total\_fac6}}$	-0.0643	0.024	-2.68
	$\beta_{\texttt{twinner:births\_total\_fac7}}$	-0.06	0.0239	-2.52
	$\beta_{\texttt{twinner:births\_total\_fac8}}$	-0.081	0.0245	-3.31
	$\beta_{\texttt{twinner:births\_total\_fac9}}$	-0.0893	0.0255	-3.5
	$\beta_{\texttt{twinner:births\_total\_fac10+}}$	-0.0719	0.024	-3
random effects	variance between `pop`	0.00111		
response family	negative binomial with log link			
	shape parameter	41.5		
fit info	number of model parameters	22		
	marginal log Likelihood	-113839		
	marginal AIC	227722		
	conditional AIC (cAIC)	227691		
data info	number of fitted observations (N)	21290		

Supplementary Table 8: Summary of the fit of model 8. The model fit corresponds to the fit of a model with the following formula: $\texttt{PP} \sim 1 + \texttt{poly}(\texttt{cbind}(\texttt{age}, \texttt{parity}), 5) + (1|\texttt{maternal\_id}) + (1|\texttt{pop})$. See legend of Supplementary Table 1 for more details.

Type	Variable	Value	Cond. SE	t-value
fixed effects	β_1	1.56	0.179	8.67
	$\beta_{\texttt{age}}$	-421	59.7	-7.04
	$\beta_{\texttt{age}^2}$	-185	51.8	-3.57
	$\beta_{\texttt{age}^3}$	-43.4	32.6	-1.33
	$\beta_{\texttt{age}^4}$	-15.8	14.4	-1.1
	$\beta_{\texttt{age}^5}$	2.3	9.7	0.237
	$\beta_{\texttt{parity}}$	48	97.7	0.491
	$\beta_{\texttt{age}\times\texttt{parity}}$	13240	39545	0.335
	$\beta_{\texttt{age}^2\times\texttt{parity}}$	-34019	30565	-1.11
	$\beta_{\texttt{age}^3\times\texttt{parity}}$	-330	14760	-0.0224
	$\beta_{\texttt{age}^4\times\texttt{parity}}$	-3263	6416	-0.509
	$\beta_{\texttt{parity}^2}$	-19.8	105	-0.188
	$\beta_{\texttt{age}\times\texttt{parity}^2}$	24157	38317	0.63
	$\beta_{\texttt{age}^2\times\texttt{parity}^2}$	-6236	22842	-0.273
	$\beta_{\texttt{age}^3\times\texttt{parity}^2}$	10729	8894	1.21
	$\beta_{\texttt{parity}^3}$	25.9	64.7	0.4
	$\beta_{\texttt{age}\times\texttt{parity}^3}$	-15049	19092	-0.788
	$\beta_{\texttt{age}^2\times\texttt{parity}^3}$	1073	8613	0.125
	$\beta_{\texttt{parity}^4}$	24	21.4	1.12
	$\beta_{\texttt{age}\times\texttt{parity}^4}$	-1749	5030	-0.348
	$\beta_{\texttt{parity}^5}$	-4.66	4.84	-0.962
random effects	variance between `maternal__id`	0.472		
	variance between `pop`	0.105		
response family	binomial with logit link			
fit info	number of model parameters	23		
	marginal log Likelihood	-40450		
	marginal AIC	80946		
	conditional AIC (cAIC)	80301		
data info	number of fitted observations (N)	105833		

Supplementary Table 9: Summary of the fit of model 9. The model fit corresponds to the fit of a model with the following formula: $\texttt{IBI} \sim \texttt{1} + \texttt{poly}(\texttt{cbind}(\texttt{age}, \texttt{parity}), \texttt{6}) + (\texttt{1}|\texttt{maternal\_id}) + (\texttt{1}|\texttt{pop})$. Note that the variable `IBI` fitted in the model actually corresponds to the duration of interbirth interval minus six months. This rescaling prevents numerical issues during the simulations. When this fitted model is used for predictions (in plots or to compute effect sizes), the missing six months are reintroduced to produce correct results. See legend of Supplementary Table 1 for other details.

Type	Variable	Value	Cond. SE	t-value
fixed effects	β_1	3.44	0.0578	59.6
	$\beta_{\texttt{age}}$	-63.4	21.9	-2.9
	$\beta_{\texttt{age}^2}$	39.5	19.6	2.01
	$\beta_{\texttt{age}^3}$	-31.2	13	-2.4
	$\beta_{\texttt{age}^4}$	14.9	6.31	2.37
	$\beta_{\texttt{age}^5}$	-4.98	2.42	-2.06
	$\beta_{\texttt{age}^6}$	4.01	1.01	3.95
	$\beta_{\texttt{parity}}$	120	40.7	2.96
	$\beta_{\texttt{age}\times\texttt{parity}}$	-29655	15604	-1.9
	$\beta_{\texttt{age}^2\times\texttt{parity}}$	26491	12834	2.06
	$\beta_{\texttt{age}^3\times\texttt{parity}}$	-15608	7263	-2.15
	$\beta_{\texttt{age}^4\times\texttt{parity}}$	5870	2912	2.02
	$\beta_{\texttt{age}^5\times\texttt{parity}}$	-2048	883	-2.32
	$\beta_{\texttt{parity}^2}$	75.2	54.5	1.38
	$\beta_{\texttt{age}\times\texttt{parity}^2}$	-30241	19139	-1.58
	$\beta_{\texttt{age}^2\times\texttt{parity}^2}$	24178	13414	1.8
	$\beta_{\texttt{age}^3\times\texttt{parity}^2}$	-11134	5960	-1.87
	$\beta_{\texttt{age}^4\times\texttt{parity}^2}$	3084	1618	1.91
	$\beta_{\texttt{parity}^3}$	52.6	42.1	1.25
	$\beta_{\texttt{age}\times\texttt{parity}^3}$	-17419	12995	-1.34
	$\beta_{\texttt{age}^2\times\texttt{parity}^3}$	9676	7207	1.34
	$\beta_{\texttt{age}^3\times\texttt{parity}^3}$	-2943	2067	-1.42
	$\beta_{\texttt{parity}^4}$	18.9	19	0.993
	$\beta_{\texttt{age}\times\texttt{parity}^4}$	-4622	4824	-0.958
	$\beta_{\texttt{age}^2\times\texttt{parity}^4}$	1891	1773	1.07
	$\beta_{\texttt{parity}^5}$	7.79	5.26	1.48
	$\beta_{\texttt{age}\times\texttt{parity}^5}$	-1409	970	-1.45
	$\beta_{\texttt{parity}^6}$	1.67	1.04	1.6
random effects	variance between `maternal_id`	0.163		
	variance between `pop`	0.00369		
response family	negative binomial with log link			
	shape parameter	5.2		
fit info	number of model parameters	31		
	marginal log Likelihood	-337277		
	marginal AIC	674616		
	conditional AIC (cAIC)	663064		
data info	number of fitted observations (N)	84543		

Supplementary Table 10: Summary of the fit of model 10. The model fit corresponds to the fit of a model with the following formula: $\texttt{T} \sim \texttt{1} + (\texttt{1}|\texttt{maternal\_id}) + (\texttt{1}|\texttt{pop})$. See legend of Supplementary Table 1 for more details.

Type	Variable	Value	Cond. SE	t-value
fixed effects	β_1	-4.06	0.0922	-44
random effects	variance between `maternal_id`	0.473		
	variance between `pop`	0.0607		
response family	binomial with logit link			
fit info	number of model parameters	3		
	marginal log Likelihood	-8911		
	marginal AIC	17828		
	conditional AIC (cAIC)	17792		
data info	number of fitted observations (N)	105833		

Supplementary Table 11: Summary of the fit of model 11. The model fit corresponds to the fit of a model with the following formula: $\texttt{T} \sim \texttt{1} + (\texttt{1}|\texttt{pop})$. See legend of Supplementary Table 1 for more details.

Type	Variable	Value	Cond. SE	t-value
fixed effects	β_1	-4.05	0.0911	-44.4
random effects	variance between pop	0.0593		
response family	binomial with logit link			
fit info	number of model parameters	2		
	marginal log Likelihood	-8927		
	marginal AIC	17857		
	conditional AIC (cAIC)	17841		
data info	number of fitted observations (N)	105833		

Supplementary Table 12: Summary of the fit of model 12. The model fit corresponds to the fit of a model with the following formula: $\texttt{T} \sim \texttt{1} + \texttt{poly}(\texttt{cbind}(\texttt{age}, \texttt{parity}), \texttt{3}) + (\texttt{1}|\texttt{pop})$. See legend of Supplementary Table 1 for more details.

Type	Variable	Value	Cond. SE	t-value
fixed effects	β_1	-4.08	0.111	-36.8
	$\beta_{\texttt{age}}$	71	30.7	2.32
	$\beta_{\texttt{age}^2}$	-60.7	17	-3.57
	$\beta_{\texttt{age}^3}$	-47.2	16.3	-2.9
	$\beta_{\texttt{parity}}$	1.54	39.4	0.0392
	$\beta_{\texttt{age}\times\texttt{parity}}$	-4468	11667	-0.383
	$\beta_{\texttt{age}^2\times\texttt{parity}}$	7856	8568	0.917
	$\beta_{\texttt{parity}^2}$	19.3	26.7	0.721
	$\beta_{\texttt{age}\times\texttt{parity}^2}$	-4441	7983	-0.556
	$\beta_{\texttt{parity}^3}$	-18.8	13.9	-1.35
random effects	variance between pop	0.0557		
response family	binomial with logit link			
fit info	number of model parameters	11		
	marginal log Likelihood	-8844		
	marginal AIC	17711		
	conditional AIC (cAIC)	17695		
data info	number of fitted observations (N)	105833		

Supplementary Table 13: Results of the goodness-of-fit tests. P-values underlined denote scenario simulations generating data for which the relationship between twinning propensity and fertility is similar to the one estimated on the raw data, using a threshold of 0.05. The two columns for p-values correspond, respectivelly from left to right, to p-values obtained in the case of the double-bootstrap or single-level bootstrap procedure (see Supplementary Notes & Supplementary Figure 7).

Scenario	GOF p-value	Raw p-value
P	<u>0.065</u>	<u>0.080</u>
I	0.005	0.005
S	0.005	0.015
H	0.005	0.005
PI	<u>0.070</u>	<u>0.090</u>
PS	<u>0.060</u>	<u>0.348</u>
PH	0.045	<u>0.060</u>
IS	0.005	0.015
IH	0.005	0.005
SH	0.005	0.015
PIS	<u>0.169</u>	<u>0.383</u>
PIH	0.035	0.045
PSH	<u>0.124</u>	<u>0.373</u>
ISH	0.005	0.015
PISH	<u>0.075</u>	<u>0.313</u>
0	0.005	0.005

Supplementary Table 14: Details of data used in the present study, for each population separately and for all populations combined. This table is the same as Table 1 from main text, but here we also include data from families with missing birth month information. Therefore this table includes an entry for the Norway dataset, in which birth month information was never available. All references are cited in main text.

Population	Locations	Maternal birth period	Mothers	Non-twinners	Twinners	Twinner rate (‰)	Offspring birth period	Births	Singleton births	Twin births	Twinning rate (‰)	Total births (min-median-max)	References
Finland East	Jaakkima, Rautu	1733-1899	911	815	96	105.38	1758-1940	4665	4562	103	22.08	1-7-17	Pettay et al. 2016; Pettay et al. 2018
Finland Lapland	Inari, Enontekiö and Sodankylä	1700-1884	757	695	62	81.90	1725-1918	3548	3482	66	18.60	1-6-13	Helle 2019
Finland SW-Archipelago	Hiittinen, Kustavi, Rymättylä	1709-1899	2795	2489	306	109.48	1732-1942	12458	12124	334	26.81	1-6-15	Haukioja et al. 1989; Lummaa et al. 1998
Finland West	Ikaalinen, Pulkkila, Tyrvää	1700-1899	5890	5396	494	83.87	1721-1943	32029	31495	534	16.67	1-7-18	Pettay et al. 2016; Pettay et al. 2018
Krummhörn	Lower Saxony, Germany	1705-1823	3739	3461	278	74.35	1725-1868	17634	17336	298	16.90	1-6-17	Gabler and Voland 1994
Norway	Smøla and Soknedal	1670-1878	1672	1556	116	69.38	1700-1915	8410	8284	126	14.98	1-6-14	Skjærvø et al. 2009
Sami Lapland	Inari, Enontekiö and Sodankylä	1703-1880	957	885	72	75.24	1729-1920	4858	4780	78	16.06	1-7-13	Helle et al. 2004; Helle 2019
Sweden Lapland	Karesuando, Jukkasjärvi, Jokkmok, Vilhelmina and Jällivaara	1721-1878	1943	1797	146	75.14	1749-1902	11106	10946	160	14.41	1-8-17	Sköld and Axelsson 2008; Sköld et al. 2011; Helle 2019
Switzerland	Linthal, Elm	1700-1899	4617	4394	223	48.30	1720-1945	21374	21138	236	11.04	1-6-18	Evans et al. 2018
All the above	All the above	1670-1899	23281	21488	1793	77.02	1700-1945	116082	114147	1935	16.67	1-7-18	This paper

Supplementary References

1. Cox, D. R. & Hinkley, D. V. *Theoretical Statistics* (Chapman & Hall, London, 1974).
2. Beran, R. Prepivoting test statistics: a bootstrap view of asymptotic refinements. *J. Am. Stat. Assoc.* **83,** 687–697 (1988).
3. Davison, A. C. & Hinkley, D. V. *Bootstrap Methods and Their Applications* (Cambridge University Press, Cambridge, 1997).
4. Efron, B. More efficient bootstrap computations. *J. Am. Stat. Assoc.* **85,** 79–89 (1990).
