# Supplementary material for: Mothers with higher twinning propensity had lower fertility in pre-industrial Europe
Source: Nat Commun. 2022 May 24;13:2886. doi: 10.1038/s41467-022-30366-9 (PMC9130277; doi:10.1038/s41467-022-30366-9)
Supplement: Supplementary file 3 — Description of Additional Supplementary Files [file 41467_2022_30366_MOESM3_ESM.pdf]

Description of Additional Supplementary Files

File Name: Supplementary Data 1

Description: Raw birth-level data used for the study. The table contains 116,082 rows (+ one for the headers) and 7 columns. Each row corresponds to a distinct birth event. The column “pop” gives the population to which the mother belonged. The column “maternal_id” gives the ID of the mother, the column “maternal_birthyear” gives the birth year of the mother. The column “maternal_age” gives the age of the mother at the focal birth event (in months). The column “birth_year” gives the year during which the birth occurred. The column “twin” indicates if the focal birth corresponded to twins (TRUE) or to a singleton (FALSE). The column “monthly” indicates if the underlying resolution of the data was originally recorded at the monthly level (TRUE) or at the yearly level (FALSE). Values labeled as NA correspond to missing data.
